# Myosin Va interacts with the exosomal protein spermine synthase

**DOI:** 10.1042/BSR20182189

**Published:** 2019-03-01

**Authors:** Luciano G. Dolce, Rui M. P. Silva-Junior, Leandro H. P. Assis, Andrey F. Z. Nascimento, Jackeline S. Araujo, Ingrid P. Meschede, Enilza M. Espreafico, Priscila O. de Giuseppe, Mário T. Murakami

**Affiliations:** 1 Graduate Program in Functional and Molecular Biology, Institute of Biology, University of Campinas, Campinas, São Paulo, Brazil; 2 Brazilian Biosciences National Laboratory (LNBio), Brazilian Center for Research in Energy and Materials (CNPEM), Zip Code 13083-970, Campinas, São Paulo, Brazil; 3 Brazilian Bioethanol Science and Technology Laboratory (CTBE), Brazilian Center for Research in Energy and Materials (CNPEM), Zip code 13083-970, Campinas, São Paulo, Brazil; 4 Department of Cell and Molecular Biology, Faculty of Medicine of Ribeirão Preto, University of São Paulo, Ribeirão Preto, São Paulo, Brazil; 5 Brazilian Synchrotron Light Laboratory (LNLS), Brazilian Center for Research in Energy and Materials (CNPEM), Campinas, Zip code 13083-970, São Paulo, Brazil

**Keywords:** cellular localization, exocytosis, myosins, protein-protein interactions, trafficking, transcription

## Abstract

Myosin Va (MyoVa) is an actin-based molecular motor that plays key roles in the final stages of secretory pathways, including neurotransmitter release. Several studies have addressed how MyoVa coordinates the trafficking of secretory vesicles, but why this molecular motor is found in exosomes is still unclear. In this work, using a yeast two-hybrid screening system, we identified the direct interaction between the globular tail domain (GTD) of MyoVa and four protein components of exosomes: the WD repeat-containing protein 48 (WDR48), the cold shock domain-containing protein E1 (CSDE1), the tandem C2 domain-containing protein 1 (TC2N), and the enzyme spermine synthase (SMS). The interaction between the GTD of MyoVa and SMS was further validated *in vitro* and displayed a *K*_d_ in the low micromolar range (3.5 ± 0.5 µM). SMS localized together with MyoVa in cytoplasmic vesicles of breast cancer MCF-7 and neuroblastoma SH-SY5Y cell lines, known to produce exosomes. Moreover, *MYO5A* knockdown decreased the expression of *SMS* gene and rendered the distribution of SMS protein diffuse, supporting a role for MyoVa in SMS expression and targeting.

## Introduction

Class V myosins are processive motors that transport or tether vesicles, organelles, and macromolecules to actin filaments, and play key roles in synaptic transmission, hormone secretion, and plasma membrane homeostasis [1–6]. They can be divided into four major structural domains with specific functions: the motor domain, which contains the actin-binding site and displays ATPase activity, the lever arm that provides a large powerstroke to occur after ATP hydrolysis, the rod region, responsible for dimerization, and the globular tail domain (GTD), a protein-binding module that allows multiple roles for these molecular motors [3,4,7–10]. In humans, three paralogous genes encode for class V myosins: *MYO5A, MYO5B*, and *MYO5C*. Defects in the *MYO5A* gene are related to the Griscelli syndrome type 1 (also known as Elejalde syndrome) [11,12], which is characterized by partial albinism and severe neurological disorders. The molecular mechanism behind the partial albinism involves defects on a tripartite complex between myosin Va (MyoVa), melanophilin, and Rab27a for melanosomes transport [13,14]. However, for the pleiotropic effects of *MYO5A* mutation in neurodevelopment, the mechanisms are still poorly understood [15].

In neuronal cells, MyoVa has been associated with organelle transport, mRNA trafficking and exocytosis of secretory vesicles [1,2]. Particularly in exocytosis, MyoVa seems to play several roles, including the capture and transport of the secretory granules in the F-actin-rich cortex, the remodeling of their membranes required for maturation, and their controlled release [16]. To date, most of the studies about MyoVa have focused on understanding how it regulates the trafficking of secretory vesicles. However, none of them have investigated whether MyoVa could also influence the internal composition of such vesicles, even though MyoVa is recurrently found in extracellular vesicles called exosomes [17].

In this work, to investigate whether MyoVa directly interacts with soluble protein components of exosomes, we performed a two-hybrid screening using the GTD of MyoVa as bait and a universal human-normalized library as prey. As envisaged, we identified the interaction of MyoVa-GTD with four proteins that compose exosomes, including the enzyme spermine synthase (SMS), which plays key roles in neurodevelopment and brain function. SMS interacts with MyoVa-GTD *in vitro*, with a *K*_d_ in the low micromolar range, and localizes together with MyoVa in vesicles at the cytoplasm of two exosome-producer cell types. *MYO5A* gene silencing led to a diffuse distribution of SMS, indicating a novel role of MyoVa in the targeting of this enzyme to secretory vesicles. Moreover, either *MYO5A* knockdown or knockout decreased *SMS* gene expression, supporting that MyoVa may influence the synthesis or stability of *SMS* mRNA. As SMS produces the neuromodulator spermine, which is stored in secretory vesicles and released via exocytosis, our findings might have implications not only in the targeting of spermine synthase to exosomes but also in the molecular mechanisms underlying the secretion of spermine.

## Materials and methods

### Yeast two-hybrid screening

The yeast two-hybrid screening was performed using the Matchmaker^®^ Gold yeast two-hybrid system (Takara Bio – Clontech) according to the manufacturer’s instructions. All reagents used in this screen was purchased from Takara Bio – Clontech, unless stated otherwise. The MyoVa-GTD-S1651E/S1652E [9] coding sequence (residues 1448–1855; NP_000250.3) was subcloned into the *EcoR*I and *Sal*I sites of pGBKT7 (pGBKT7-EE construct), and was used to transform *Saccharomyces cerevisiae* Y2H Gold® strain. This phosphomimetic (EE) construct was the bait because our original aim was to identify phospho-specific interactions. The pGADT7-Prey plasmid of clones positive for the activation of all reporter genes of the system (*HIS3, ADE2, AUR1-C*, and *MEL1*) were extracted using the Easy Yeast Plasmid Isolation Kit. The purified vectors were transformed into *Escherichia coli* DH5α competent cells, extracted with the QIAprep Spin Miniprep Kit (Qiagen), submitted to DNA sequencing, and compared with non-redundant sequence databases using BLASTn and BLASTx [18].

### Yeast two-hybrid pairwise validation

To validate the positive hits identified in the yeast two-hybrid screen and to test whether the interactions were dependent of the phosphomimetic mutation, *S. cerevisiae* Y2H Gold^®^ cells were co-transformed with a pair of pGBKT7 and pGADT7 vectors (Supplementary Table S1), and grown at 30°C for 4 days on QDO/X/A-agar plates – a synthetic defined agar medium without tryptophan, leucine, histidine and adenine, and supplemented with 200 ng/ml aureobasidin A and 40 μg/ml 5-bromo-4-chloro-3-indolyl alpha-D-galactopyranoside (Takara Bio – Clontech). To remove potential false-positive results, we tested the activation of all reporter genes in Y2H Gold^®^ cells co-transformed with pGADT7-Prey and empty pGBKT7 vectors (negative controls).

### Bioinformatics validation

The nucleotide sequences of preys validated in the previous step were subjected to bioinformatics analyses to filter out possible false positives still present. Initially, the sequences were analyzed using BLASTn [18] to remove those hits containing 5′ or 3′ UTR in frame with the GAL4 AD sequence, which would generate artificial fusion proteins. For constructs containing truncated open reading frames (ORF), only those hits containing at least one intact domain were considered as true positives, according to protein sequence analyses using the SMART server [19].

### Molecular cloning

The full-length *SMS* ORF (NM_004595.4, 253-1353 pb) was amplified from a human cDNA library by PCR using the primers shown in Supplementary Table S2. The PCR product was purified with QIAquick PCR Purification Kit (Qiagen), digested using *Nde*I and *Xho*I restriction enzymes and inserted into the *Nde*I/*Xho*I sites of pET28a tobacco etch virus (TEV) vector [9] using T4 DNA Ligase (Promega). Positive clones were confirmed by DNA sequencing.

### Microscale thermophoresis

To perform the microscale thermophoresis (MST) experiment, the wild-type MyoVa-GTD [9] and SMS constructs (both in pET28a-TEV vector) were produced in BL21(DE3)∆SlyD strain containing the plasmid pRARE2, in LB medium [20] at 25°C for 4 h, and 20°C for 16 h, respectively. The cell lysis was performed by sonication (Vibra-cells, Sonics) with lysis buffer (50 mM HEPES, 500 mM NaCl, 5% *(v/v)* glycerol, 20 mM imidazole, pH 7.4) supplemented with 0.1 mg/ml lysozyme, and SIGMAFAST™ Protease Inhibitor Tablets (Sigma-Aldrich). All proteins were purified by two chromatographic steps, using a HiTrap Chelating column (GE Healthcare), with an imidazole gradient for protein elution, and a HiLoad Superdex 200 (or 75) 16/60 column (GE Healthcare). The size-exclusion chromatography (SEC) was carried out in SEC buffer (20 mM HEPES, 150 mM NaCl, 5% *(v/v)* glycerol, pH 7.4). MyoVa-GTD purified by affinity chromatography was treated with a His-tagged TEV protease [21] (4°C, 20 h) for the cleavage of the 6xHis-tag before the purification in a HiLoad Superdex 200 16/60 column (GE Healthcare) pre-equilibrated with SEC buffer.

The MST experiment was performed using a Monolith™ NT.115 (NanoTemper Technologies) device, with a LED power of 40%, and a MST power of 60%. SMS was labeled with the His-Tag Labeling Kit RED-tris-NTA (NanoTemper Technologies), following the manufacturer’s labeling protocol, and loaded into Monolith™ NT.115 MST premium-coated capillaries (NanoTemper Technologies). All assays were performed in triplicate using 50 nM His-tagged-labeled-SMS and a serial dilution of MyoVa-GTD to the maximum possible concentration in SEC buffer. The differences between the cold and hot states of each of the 16 MST profiles were used to determine the change in fluorescence intensities for each profile using the following equation: $F_{norm}=F_{hot}/F_{cold}\cdot 1000$, where F is the fluorescence measured in each state. The data were processed using the NTAffinity Analysis software (NanoTemper Technologies) and the mean of *F_norm_* triplicates plotted against ligand concentration were fitted with the Hill equation ($F_{norm}={\left[L\right]}^{n}/K_{d}+{\left[L\right]}^{n}$, where [L] = ligand concentration, n = Hill coefficient, and *K*_d_ = dissociation constant) using the Origin 8.0 software to estimate the *K*_d_.

### Immunocytochemistry and gene silencing assays

Immunocytochemistry assays were performed on human neuroblastoma (SH-SY5Y) and human mammary adenocarcinoma (MCF-7) cell lines cultured in Dulbecco’s modified Eagle’s medium (DMEM) with high glucose (GIBCO - Thermo Fisher Scientific: 12800-017) supplemented with 10% *(v/v)* fetal bovine serum (FBS) 100 units/ml penicillin and 100 μg/ml of streptomycin and kept in a humid atmosphere containing 5% *(v/v)* CO_2_ at 37°C. The Stealth RNAi™ siRNAs targeting *MYO5A* were purchased from Invitrogen – Thermo Fisher Scientific and are shown in Supplementary Table S3. A scramble sequence Stealth RNAi™ siRNA Negative Control High GC Duplex (Invitrogen – Thermo Fisher Scientific) was used as a control.

MCF-7 cells were transfected using DharmaFECT 1 Transfection Reagent (GE Healthcare) according to the manufacturer’s instructions. Cells were fixed with 2% *(v/v)* paraformaldehyde pH 7.4 for 20 min, and then permeabilized with 0.3% *(v/v)* Triton X-100, blocked with 100 mM glycine, and then 3% *(m/v*) BSA (adapted from Assis et al. [7]). The following antibodies were used: 1 μg/ml rabbit anti-SMS (Sigma-Aldrich: HPA029852); 2 μg/ml rat polyclonal affinity-purified anti-MyoVa_Medial_Tail (*in house*, manuscript in preparation). Secondary antibodies used were 2 μg/ml Alexa Fluor^®^ goat anti-rat 488 (Abcam: ab150157), 2 μg/ml Alexa Fluor^®^ donkey anti-rabbit 594 (Molecular Probes: A21207) IgG. The slides were mounted on ProLong^®^ Diamond Antifade Mountant medium with DAPI (Thermo Fisher Scientific: P36962) and the images were collected on Zeiss LSM780 Axio Observer multifotons inverted confocal microscope, with a 63× objective. The Icy BioImage open source software from the Pasteur Institute (http://icy.bioimageanalysis.org/) was used for image processing and calculation of the Pearson’s coefficients with the colocalization studio plugin [22]. All the compared images were acquired with the same parameters. The raw files were used for all quantifications.

### RNA isolation and quantitative PCR

Total RNA was extracted from the cells samples according to standard TRIzol protocol (Invitrogen – Thermo Fisher Scientific). Total RNA (1 μg) was reverse transcribed to cDNA using High Capacity cDNA Reverse Transcription Kit (Applied Biosystems – Thermo Fisher Scientific) according to a standard manufacturer’s protocol followed by amplification on the ABI 7500 Real-Time PCR System (Applied Biosystems – Thermo Fisher Scientific), using primers from the Supplementary Table S4. Samples of SH-SY5Y cells (control and differentiated) were prepared as described below. All quantitative PCR (qPCR) analyses were performed in triplicate. The expression of endogenous control (GAPDH) was used for the normalization of RNA input.

Gene expression levels were calculated by relative quantitation using the ABI 7500 Real-Time PCR SDS 1.2 software (Applied Biosystems – Thermo Fisher Scientific) and the fold expression changes were determined by 2^−ΔΔC^_T_ method [23]. The data are presented as the fold change of mRNA expression in cells treated with siRNA relative to cells treated with siControl after normalization to an endogenous control (GAPDH or TBP). The qPCR data were described as mean ± standard deviation and analyzed using the Student’s *t*test, with *P*≤0.01 considered statistically significant.

### Neuronal differentiation

SH-SY5Y cells were cultivated in DMEM high glucose (GIBCO – Thermo Fisher Scientific: 12800-017), supplemented with 10% (v/v) FBS (GIBCO – Thermo Fisher Scientific) and 100 units/ml penicillin and 100 μg/ml of streptomycin (GIBCO – Thermo Fisher Scientific: 15140122) and kept in a humid atmosphere containing 5% (v/v) CO_2_ at 37°C. Cellular differentiation was induced as described by Encinas and coworkers [24] with some modifications. Briefly, 2.5 × 10^4^ cells/cm^2^ were plated on DMEM high glucose medium supplemented with 10% (v/v) FBS and 1% penicillin/streptomycin solution, in culture plates or on 13 mm^2^ glass coverslips previously treated with 0.1 mg/ml poly-D-lysin (Sigma-Aldrich). The next day, the medium was removed and replaced with medium II (DMEM high glucose medium containing 1% (v/v) FBS, 1% penicillin/streptomycin solution and 10 μM retinoic acid [Abcam: ab120728]). After 3 days of growth, medium was replaced with fresh medium II supplemented with 50 ng/ml brain-derived neurotrophic factor (BDNF; Sigma-Aldrich: SRP3014) and cells were grown for further 4 days. RNA extraction and qPCR assays were performed as described above.

### Culture of FO and RO fibroblasts

FO cells are human fibroblasts isolated from skin fragments of a patient carrying a mutation in the *MYO5A* gene, which renders these cells null for MyoVa protein (Griscelli Syndrome type I/Elejalde syndrome). RO cells are normal human fibroblasts age-paired with FO, also isolated in the same laboratory, and used as control. The cells were cultured in DMEM high glucose (GIBCO – Thermo Fisher Scientific: 12800-017) supplemented with 10% (*v/v*) FBS (GIBCO – Thermo Fisher Scientific), at 37°C, 5% (*v/v*) CO_2_. Total RNA was extracted using standard TRIzol protocol and used for cDNA synthesis and qPCR, as described above. All methods presented here were performed in accordance with the relevant guidelines and regulations approved by Faculty of Medicine of Ribeirão Preto, University of São Paulo and in accordance with The Code of Ethics of the World Medical Association (Declaration of Helsinki). Primary cultures were established with the informed consent of the patients and with the approval of the Research Ethics Committee of the Faculty of Medicine of Ribeirão Preto.

## Results

**Figure 1:**
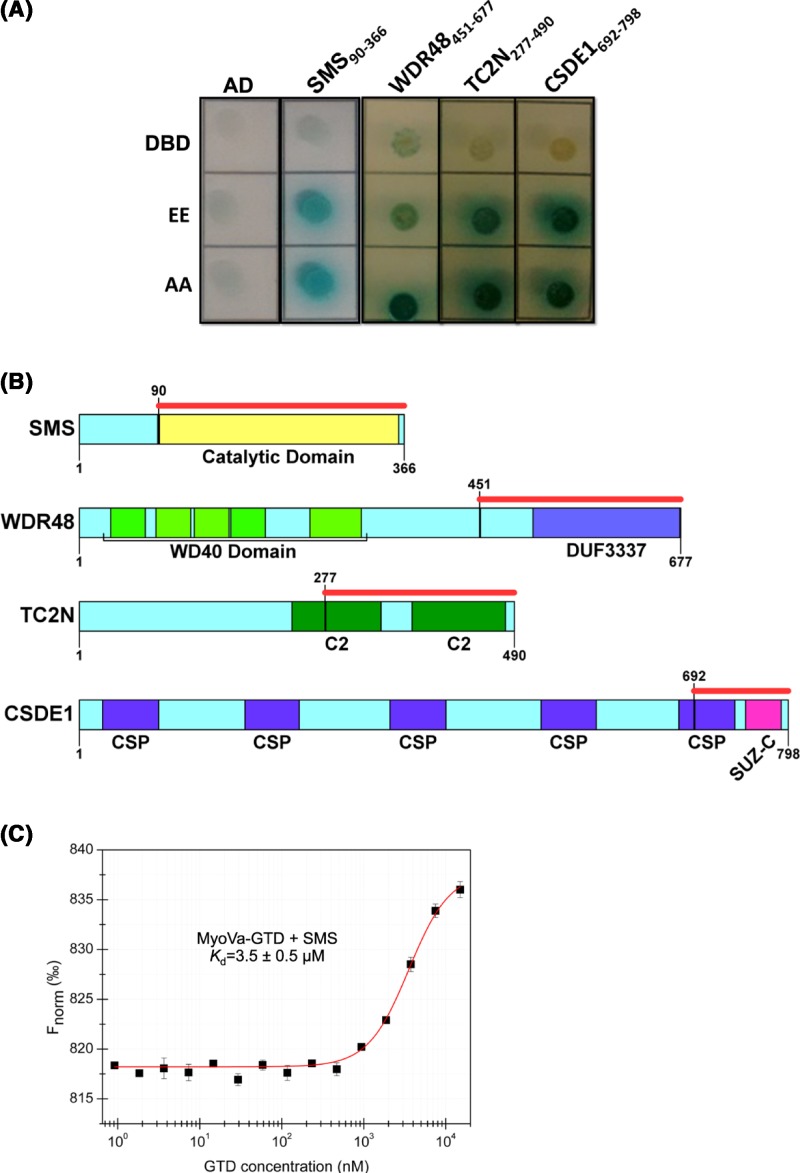
Yeast two-hybrid and *in vitro* assays reveal SMS as a novel binding partner of MyoVa-GTD (**A**) Pairwise two-hybrid assays showing the activation of four reporter genes (QDO/X/A selective medium) in yeast cells co-transformed with pGADT7-prey (or empty pGADT7 as a control) plasmids and pGBKT7-GTD constructs (EE = phosphomimetic and AA = non-phosphorylated) showing that the identified interactions are not specific to the phosphomimetic mutant. The first line represents the negative control assays, where each clone was tested against the empty pGBKT7 vector (DBD). (**B**) Schematic representation of the domain architecture of the new-found partners. The red bar indicates the prey boundaries found in the yeast two-hybrid screening: SMS^90–366^ = catalytic domain of SMS (NCBI accession number: EAW98985); WDR48^451–677^ = DUF3337 domain of the WDR48 (NCBI accession number EAW64548); TC2N^277–490^ = contains the second C2 domain of the tandem C2 domains nuclear protein (TC2N; NCBI accession number: EAW81464); CSDE1^692–798^ = contains the SUZ-C domain of the cold shock domain-containing protein E1, isoform 4 (CSDE1; NCBI accession number: NP_001007554). Figures were made using SMART [19] and IBS [41]. (**C**) MST assays showing that MyoVa-GTD binds to SMS with a *K*_d_ in the low micromolar range. F_norm_ = normalized fluorescence. Data are presented as mean ± SD (error bars) from triplicates.

Using MyoVa-GTD as bait, we obtained 54 clones in a yeast two-hybrid screen of a human-normalized cDNA library. These clones represented 35 different genes, according to DNA sequence analyses of the prey plasmids. To further validate these results, we performed a pairwise two-hybrid assay resulting in 21 positive clones. However, nine of them were from noncoding regions, such as 5′ and 3′ UTRs of mRNA, and three encoded zinc finger proteins or heat shock proteins, which are recurrent false positives in yeast two-hybrid assays [25] (Table 1). After the exclusion of truncated single-domain proteins (probably misfolded), four clones remained as potential binding partners of MyoVa-GTD: SMS, WDR48, CSDE1, and TC2N (Figure 1A,B). Interestingly, these four proteins are known components of exosomes (Table 2). Further, we evaluated whether these interactions could be regulated by the MyoVa-GTD phosphorylation at Ser1652. However, the four proteins interacted with both the GTD-EE (S1651E/S1652E) and GTD-AA (S1651A/S1652A) constructs in a pairwise two-hybrid assay, indicating that their binding to GTD is independent of the phosphorylation under the tested conditions (Figure 1A). The Ala mutant was used to mimic the nonphosphorylated state of MyoVa-GTD and preserves the structure and dynamic behavior of the wild-type construct produced in *E. coli* [9].

**Table 1:** Positive results of pairwise yeast two-hybrid assays curated by bioinformatic analyses

Clone	Blastn result	Prey interval aligned to the mRNA (pb)	mRNA translation interval (pb)	Prey interval aligned to the polypeptide (aa)	Protein name (Length)	Domain composition (Position)	Description
**2**	NM_005180.8	1084–2167	507–1487				ZnF – Recurrent false positive
**4**	XM_011519161.1	88–1216	4739–7036				5’ UTR
**6**	NM_001306191.1	793–1779	242–637				3’ UTR
**7**	NM_006016.4	1549–1724	182–775				3’ UTR
**16**	BC050683.1	302–1241	451–1887				ZnF – Recurrent false positive
**19**	NM_018442.3	2122–2736	354–2996	589–794*	*DDB1 and CUL4 associated factor 6* (880)	Several WD40 repeat domains	Probably misfolded protein
**25**	AC002549.1	92606–91778					Chromosomal untranscribed regions
**30**	**NM_020839.3**	**1395–2380**	**41–2074**	**451–677**	***WD repeat-containing protein 48* (677)**	**WD40 (20–390) e DUF 3337 (509–674)**	**Regulator of deubiquitinating complexes** [42]
**36**	AL049695.20	39457–40568					Chromosomal untranscribed regions
**44**	BC016058.1	810–1785	230–1219	193–329	*Cathepsin K* (329)	I29 (26–86) e Pept_C1 (105–320)	Probably misfolded protein
**49**	BC000006.2	584–1255	122–1033	154–303	*ATPase, Na+/K+ transporting, beta 1 polypeptide* (303)	Na_K-APTase (3–297)	Probably misfolded protein
**68**	NM_001165979.2	7561–7955	635–7495				3’ UTR
**78**	NG_008805.2	8745–9860	396–9011	2783–2871	*Fibrillin 1* (2871)		Unfolded region
**88**	NR_033192.1	198–1074	45-774				HSP – Recurrent false positive
**104**	BC038384.1	894–1513	121–1095	258–324	*Y box binding protein 1* (324)	CSP (60–128)	Unfolded region
**106**	NM_138773.2	3372–4495	127–1383				3’ UTR
**108**	AL139288.15	138495–139600					Chromosomal untranscribed regions
**123**	**NG_009228.1**	**293–831**	**23–1129**	**90–269** *	***Spermine synthase* (366)**	**Spermine_synth (89–366)**	**Converts spermidine into spermine** [32]
**142**	**NM_152332.5**	**1045–2180**	**214–1686**	**277–490**	***Tandem C2 domains nuclear protein* (490)**	**C2 (240–340) e C2 (375–480)**	**Synaptotagmin-like tandem C2 protein**
**151**	XM_006716253.2	4257–5384	115–972				3’ UTR
**186**	**NM_001007553.2**	**2590–3749**	**514–2910**	**692–798**	***Cold shock domain-containing protein E1* (798)**	**CSP (26–89), CSP (190–250), CSP (350–420), CSP (520–590), CSP (675–740) e SUZ-C (750–790)**	**RNA-binding protein** [43]

Potential MyoVa-binding partners are highlighted in bold.

* The final pb identified in our sequencing, not the final pb in the prey.

**Table 2:** Proteins that interacted with MyoVa-GTD in the yeast two-hybrid system are components of exosomes and/or secretory vesicles

Protein	Tissue/cell type [vesicle type]	Database [ID]	Ref.
SMS	Breast cancer cells [exosomes]	Vesiclepedia [VP_20603]	[44]
WDR48	Breast cancer cells [exosomes]; melanoma cells [extracellular vesicles];	Vesiclepedia [VP_67561]	[44]
	Mouse embryonic fibroblasts [extracellular vesicles]		
TC2N	Breast milk [exosomes]; colorectal cancer cells [microvesicles];	Vesiclepedia [VP_123036]	[44]
	Mesenchymal stem cells [microvesicles]; urine [exosomes]		
CSDE1	Mast cells [exosomes]	Exocarta [ExoCarta_229663]	[17]

After analyzing the functional data available for SMS, WDR48, CSDE1, and TC2N in the literature, we decided to further characterize the interaction between MyoVa-GTD and SMS due to the key role of this enzyme in neurodevelopment [26,27]. For *in vitro* affinity assays, MyoVa-GTD and SMS proteins were expressed and purified to homogeneity (Supplementary Figures S1 and S2), the His-tag of MyoVa-GTD was removed, and the purified SMS protein was labeled with the RED-tris-NTA dye (Supplementary Figure S3). According to MST experiments, MyoVa-GTD and SMS formed a complex *in vitro* with a dissociation constant of 3.5 ± 0.5 µM (Figure 1C), supporting SMS as a novel MyoVa-binding partner.

**Figure 2:**
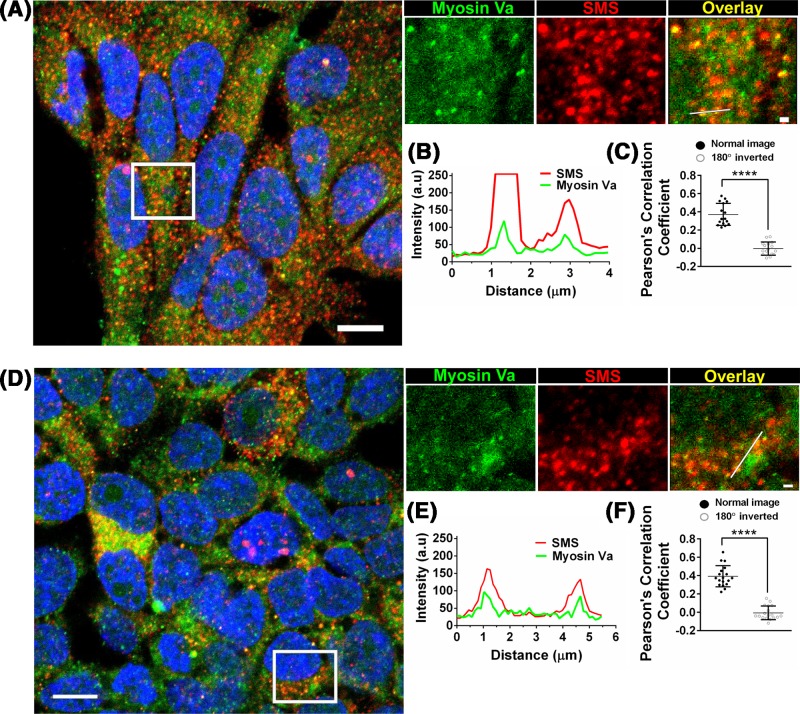
MyoVa and SMS localize together in discrete puncta at the cytoplasm Immunocytochemistry showing the colocalization of MyoVa (green; anti-MyoVa-Medial tail) and SMS (red) labeling in MCF-7 (**A-C**) and SH-SY5Y (**D-F**) cell lines. The overlap of the fluorescent signals is shown in yellow (overlay). (**B,E**) Linescan analysis of two representative colocalization sites. (**C, F**) Pearson’s correlation coefficients support the partial colocalization of MyoVa (green) and SMS (red) labeling. The correlation is based on the average of 16 and 18 independent cells, for MCF-7 and SH-SY5Y cells, respectively, with standard deviations shown. The correlation between MyoVa (rotated 180°) and SMS channels was used as negative control (random colocalization). Nucleus were stained with DAPI (Blue). Scale bar: 10 µm (field), 1 µm (zoom). **** *P*≤0.001 from two-tailed Wilcoxon’s non-parametric rank test in C (because one of the populations were not normally distributed) and two-tailed paired *t* test in F).

**Figure 3:**
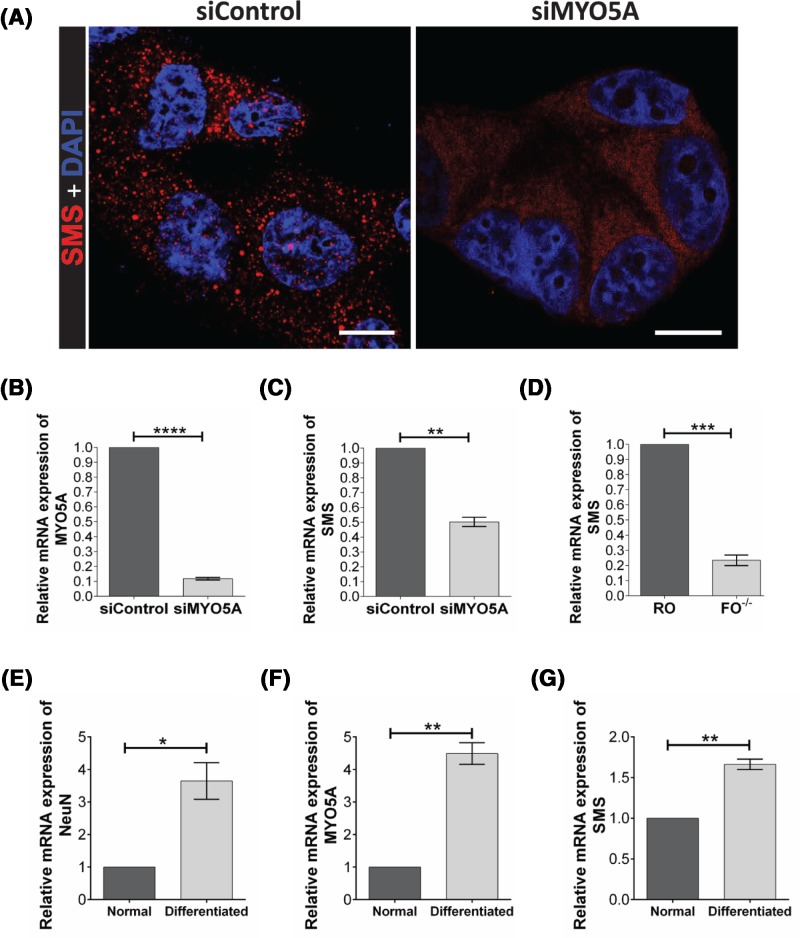
*MYO5A* knockdown affects SMS distribution and expression in MCF-7 cells (**A**) Immunocytochemistry showing that the puncta distribution of SMS (siControl) became diffuse in cells treated with *siMYO5A*. Nucleus were stained with DAPI (Blue). Scale bar: 10 µm. (**B**) qPCR showing that the *siMYO5A* treatment was effective to reduce *MYO5A* mRNA expression. (**C**) The qPCR showing the relative mRNA expression of *SMS* in cells treated with siControl or si*MYO5A* and in (**D**) *MYO5A*-null fibroblasts (FO^−/−^) and the normal control (RO). The qPCR analysis of expression of the marker for neuronal differentiation *NeuN* (**E**), *MYO5A* (**F**), and *SMS* (**G**) in normal and differentiated neuronal SH-SY5Y cells. * *P*≤0.05; ** *P*≤0.01; *** *P*≤0.001, **** *P*≤0.0001. Paired *t* test, two-tailed.

In breast cancer (MCF-7) and neuroblastoma (SH-SY5Y) cell lines, SMS localized together with a subset of MyoVa-labeled vesicles at the cytoplasm (Figure 2). We also noticed that *MYO5A* knockdown induced a more diffuse distribution of SMS, in contrast to the fewer, but sharper puncta observed at the control (Figure 3A), indicating that MyoVa may play a role in the targeting of SMS to vesicles.

According to qPCR analyses, the mRNA expression levels of *SMS* decreased upon *MYO5A*-silencing (Figure 3B,C), which correlated with a slight decrease in SMS protein content assessed by Western blot (Supplementary Figure S4). To investigate a possible correlation between depletion of MyoVa protein and *SMS* transcription, we also evaluated the expression of *SMS* in *MYO5A*-null primary fibroblasts isolated from patients with Griscelli Syndrome Type 1/Elejalde syndrome. As expected, the *SMS* expression was lower in cells lacking functional MyoVa compared with normal primary fibroblasts, indicating a role for MyoVa in the synthesis or stability of *SMS* mRNA (Figure 3D). In contrast, when the expression of *MYO5A* was stimulated upon neuron differentiation, the expression level of *SMS* also increased, further supporting a correlation between the expression of these two genes (Figure 3E–G).

## Discussion

In this work, we reveal the direct interaction between MyoVa-GTD and four components of exosomes – WDR48, CSDE1, TC2N, and SMS – using a highly stringent yeast two-hybrid system. Furthermore, we validate the interaction between MyoVa-GTD and the enzyme SMS *in vitro*, and provide primary evidence about a new role for MyoVa in the expression and targeting of SMS into secretory vesicles.

MyoVa-GTD and SMS form complexes *in vitro* with a dissociation constant in the low micromolar range (*K*_d_ = 3.5 ± 0.5 µM), which is typical of transient complexes and is similar to the affinity of MyoVa-GTD to other binding partners, such as the C2 domains of RPGRIP1L (3–9 µM, also determined by MST but using a covalent probe) [7]. The protein SMS has been found in the cytosol, in nuclear bodies and in exosomes [28–30]. This enzyme converts spermidine into spermine (EC 2.5.1.22) [31–33], a polyamine that acts as second messenger in neurotransmission, targeting receptors in postsynaptic membranes [34–36]. The vesicular storage of spermine and spermidine involves an active transporter from the SLC18 family, but the mechanisms coupling spermine synthesis to secretion are still elusive [37]. In this context, and based on our results showing the physical interaction between MyoVa-GTD and SMS, the colocalization of SMS and MyoVa in a subset of cytoplasmic vesicles and the more diffuse distribution of SMS protein upon *MYO5A* gene silencing, it is tempting to hypothesize that the actin-based motor MyoVa targets the enzyme that produces spermine to secretory vesicles for spermine secretion via exocytosis [37,38], and/or SMS release via exosomes [28,29]. Moreover, whether and how the interaction between MyoVa and SMS influence the spermine synthase activity should be further evaluated in future studies.

Besides our RNAi studies show lower levels of *SMS* mRNA upon *MYO5A* silencing, other evidences support a correlation between *MYO5A* and *SMS* expression. In *MYO5A*-null fibroblasts, *SMS* transcripts are less abundant than in normal cells, indicating that functional MyoVa is required for the usual expression of *SMS* gene. Moreover, *MYO5A* and *SMS* are up-regulated in differentiated neurons and the proteins they encode are less abundant in human brains with Huntington’s disease, a progressive neurodegenerative disorder [39]. Phenotypic traits further support a correlation between MyoVa and SMS, since patients harboring loss-of-function mutations in *SMS* or *MYO5A* genes share some neurological symptoms, including intellectual disability, seizures, and hypotonia [12,27,40].

In summary, the new interaction between MyoVa and exosomes components, including the enzyme SMS, and the co-occurrence of MyoVa and SMS in cytoplasmic vesicles bring new perspectives about the roles of this molecular motor in exocytic pathways, especially in the filling of exosomes and secretion of the neuromodulator spermine.

## Availability of materials and data

Materials, data, and associated protocols will be promptly available to readers without undue qualifications in material transfer agreements.

## Supporting information

**Figure S1.** Purification of SMS protein (43.5 kDa). **A.** Chromatogram of the affinity chromatography, showing 4 elution peaks. **B.** SDS-PAGE of the input (I) and the four peaks (1 to 4) from the affinity chromatography, stained with coomassie blue and photographed. (M: Pierce™ Unstained Protein MW Marker, Thermo Fisher Scientific) **C.** Chromatogram of the size exclusion chromatography of sample 3 in a Superdex 75 pg 16/600 column. Fractions containing SMS dimers (Ve ∼ 70 mL) were pooled and used in the MST assays.

**Figure S2.** Purification of MyoVa-GTD (50.3 kDa). **A.** Chromatogram of the affinity chromatography showing one elution peak. **B.** Chromatogram of the size exclusion chromatography of TEV-treated MyoVa-GTD using a HiLoad Superdex 200 16/60 column. **C.** SDS-PAGE of the input (1) and the elution peak (2) from the size exclusion chromatography. (M: Pierce™ Unstained Protein MW Marker, Thermo Fisher Scientific). The SDS-PAGE was silver stained and photographed.

**Figure S3.** Control experiments of MST assays. **A.** Silver-stained SDS-PAGE of (1) 6xHis-MyoVa-GTD and (2) MyoVa-GTD after incubation with TEV protease. Note the band shift indicating the removal of 6xHis tag. **B.** Titration curve of 6xHis-MyoVa-GTD (black squares), TEV-treated MyoVa GTD (red circles) and 6xHis-SMS (blue triangles) against 25 nM RED-tris-NTA dye, following manufacturer’s instructions to estimate the dye affinity to each protein. According to the Hill fit (red lines) of the 6xHis-MyoVa-GTD and 6xHis-SMS profiles, we estimated a *K*d for the dye-protein complex of 28 ± 6 nM and 6.7 ± 0.6 nM, respectively. No interaction was detected between TEV-treated MyoVa-GTD and the RED-tris-NTA dye, indicating the efficient removal of 6xHis-tag by TEV protease.

**Figure S4.** Quantification of SMS protein by western blot (A) in cells treated with siControl or siMYO5A. The graph (B) represents the quantification of the signal intensity of the bands by densitometry, using γ-tubulin as endogenous control. * *p* ≤ 0.05, Paired t-test, two-tailed.

**Table S1:** List of the pairwise validation co-transformants. pGADT7-Prey = prey plasmids identified in the screen, purified and sequenced. Ø = empty vector. pGBKT7-EE = MyoVa-GTD-S1651E/S1652E construct. pGBKT7-AA = MyoVa-GTD-S1651A/S1652A construct.

**Table S2:** Primers for amplification of SMS construct.

**Table S3:** Stealth RNAi™ siRNA targeting *MYO5A*.

**Table S4:** Primers used for qPCR assays.

## Abbreviations

AD: activation domain
DBD: DNA-binding domain
DMEM: Dulbecco’s modified Eagle’s medium
GTD: globular tail domain
MST: microscale thermophoresis
MyoVa: myosin Va
NTA: nitrilotriacetic acid
ORF: open reading frame
qPCR: quantitative PCR
SEC: size-exclusion chromatography
SMS: spermine synthase
TEV: tobacco etch virus
