# Supplementary material for: Myosin Va interacts with the exosomal protein spermine synthase
Source: Biosci Rep. 2019 Mar 1;39(3):BSR20182189. doi: 10.1042/BSR20182189 (PMC6395372; doi:10.1042/BSR20182189)
Supplement: Supplementary file 1 [file bsr-39-bsr20182189_Supp1.pdf]

**Supplementary information**

# Myosin Va interacts with the exosomal protein spermine synthase

Luciano G. Dolce[1,2,3#], Rui M. P. Silva-Junior[4#], Leandro H. P. Assis[1,2], Andrey F. Z. Nascimento[1,5], Jackeline S. Araujo[4], Ingrid P. Meschede[4], Enilza M. Espreafico[4*], Priscila O. de Giuseppe[2,3*] and Mário T. Murakami[2,3*]

**Table S1:** List of the pairwise validation co-transformants. pGADT7-Prey = prey plasmids identified in the screen, purified and sequenced. Ø = empty vector. pGBKT7-EE = MyoVa-GTD-S1651E/S1652E construct. pGBKT7-AA = MyoVa-GTD-S1651A/S1652A construct.

| Assay | Vector pair |
|---|---|
| Negative control | pGADT7Ø + pGBKT7Ø |
| Prey auto-activation control | pGADT7-Prey + pGBKT7Ø |
| Bait auto-activation control | pGADT7Ø + pGBKT7-EE or pGADT7Ø + pGBKT7-AA |
| Prey•GTD-EE interaction | pGADT7-Prey + pGBKT7-EE |
| Prey•GTD-AA interaction | pGADT7-Prey + pGBKT7-AA |

**Table S2.** Primers for amplification of SMS construct.

| Primer | Sequence |
|---|---|
| **SMS F** | AAAAGGATCCCATATGGCAGCAGCACGGCAC |
| **SMS R** | AAAACTCGAGTCAGGGTTTAGCTTTCTTCCAAACAGTG |

**Table S3.** Stealth RNAi™ siRNA targeting *MYO5A.*

| Primer | Sequence |
|---|---|
| **1** | 5′-CCAGGCCCGAUGCUAUGCUAAGUUU-3′ |
| **2** | 5′-AAACUUAGCAUAGCAUCGGGCCUGG-3′ |
| **3** | 5′-CCUUAAUGAUGAAAGGCUGAGAUAU-3′ |
| **4** | 5′-AUAUCUCAGCCUUUCAUCAUUAAGG-3′ |
| **5** | 5′-GAAAUAGCACAAGCAUACAUUGGUU-3′ |
| **6** | 5′-AACCAAUGUAUGCUUGUGCUAUUUC-3′ |

**Table S4.** Primers used for qPCR assays.

| Primer | Sequence 5`- 3` |
|---|---|
| ***MYO5A*** *H. sapiens*- F | CGGAAAGACCTGGAGCAAACTC |
| ***MYO5A*** *H.sapiens* – R | TGCTGCACGATGCGGTGATTGA |
| ***SMS*** *H. sapiens*- F | AGGAGATCGTCTGTGTCCCTTC |
| ***SMS*** *H.sapiens* – R | GGAGGTCAGGAAGGCTATTTGC |
| ***HRNBP3*** **(NeuN)** *H. sapiens* – F | TACGCAGCCTACAGATACGCTC |
| ***HRNBP3*** **(NeuN)** *H. sapiens* – R | TGGTTCCAATGCTGTAGGTCGC |

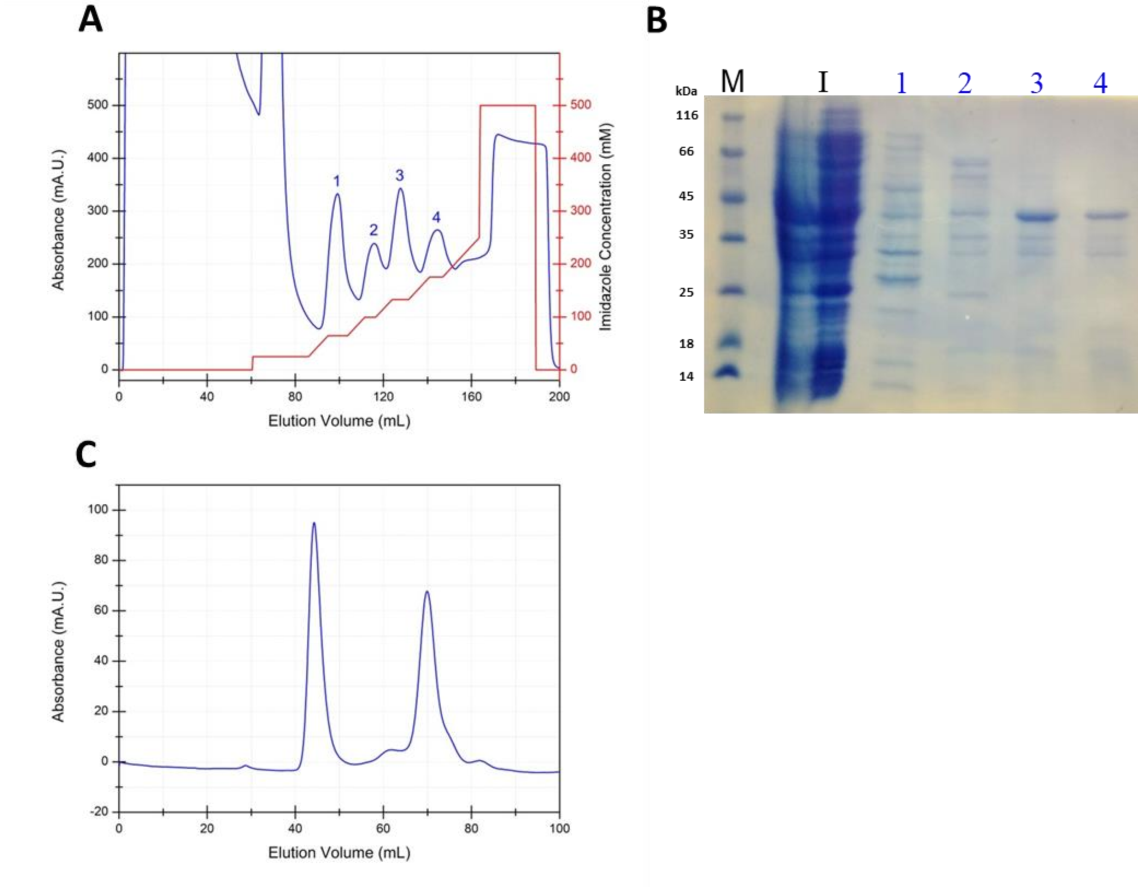


**Figure S1.** Purification of SMS protein (43.5 kDa). **A.** Chromatogram of the affinity chromatography, showing 4 elution peaks. **B**. SDS-PAGE of the input (I) and the four peaks (1 to 4) from the affinity chromatography, stained with coomassie blue and photographed. (M: Pierce™ Unstained Protein MW Marker, Thermo Fisher Scientific) **C**. Chromatogram of the size exclusion chromatography of sample 3 in a Superdex 75 pg 16/600 column. Fractions containing SMS dimers ($V_e$ ~ 70 mL) were pooled and used in the MST assays.

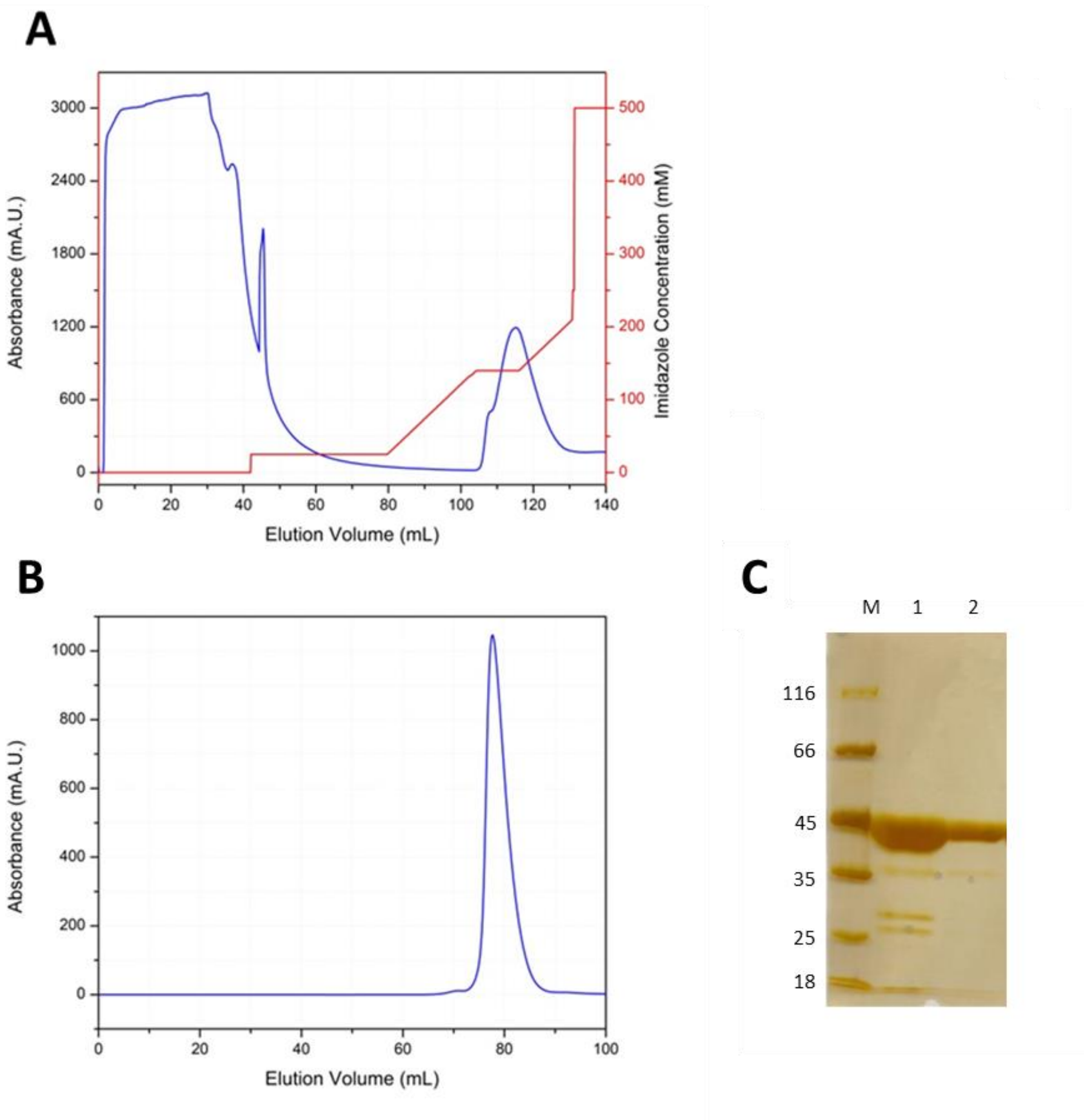


**Figure S2.** Purification of MyoVa-GTD (50.3 kDa). **A.** Chromatogram of the affinity chromatography showing one elution peak. **B.** Chromatogram of the size exclusion chromatography of TEV-treated MyoVa-GTD using a HiLoad Superdex 200 16/60 column. **C.** SDS-PAGE of the input (1) and the elution peak (2) from the size

exclusion chromatography. (M: Pierce™ Unstained Protein MW Marker, Thermo Fisher Scientific). The SDS-PAGE was silver stained and photographed.

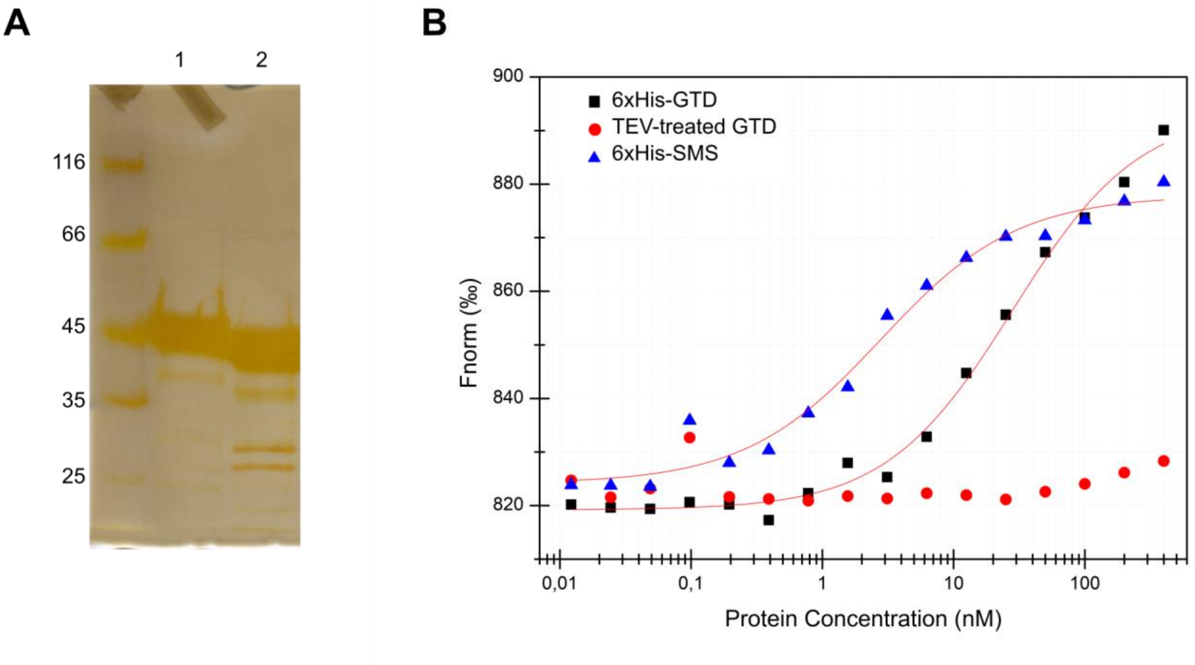


**Figure S3. Control experiments of MST assays. A.** Silver-stained SDS-PAGE of (1) 6xHis-MyoVa-GTD and (2) MyoVa-GTD after incubation with TEV protease. Note the band shift indicating the removal of 6xHis tag. **B.** Titration curve of 6xHis-MyoVa-GTD (black squares), TEV-treated MyoVa GTD (red circles) and 6xHis-SMS (blue triangles) against 25 nM RED-tris-NTA dye, following manufacturer´s instructions to estimate the dye affinity to each protein. According to the Hill fit (red lines) of the 6xHis-MyoVa-GTD and 6xHis-SMS profiles, we estimated a $K_d$ for the dye-protein complex of 28 ± 6 nM and 6.7 ± 0.6 nM, respectively. No interaction was detected between TEV-treated MyoVa-GTD and the RED-tris-NTA dye, indicating the efficient removal of 6xHis-tag by TEV protease.

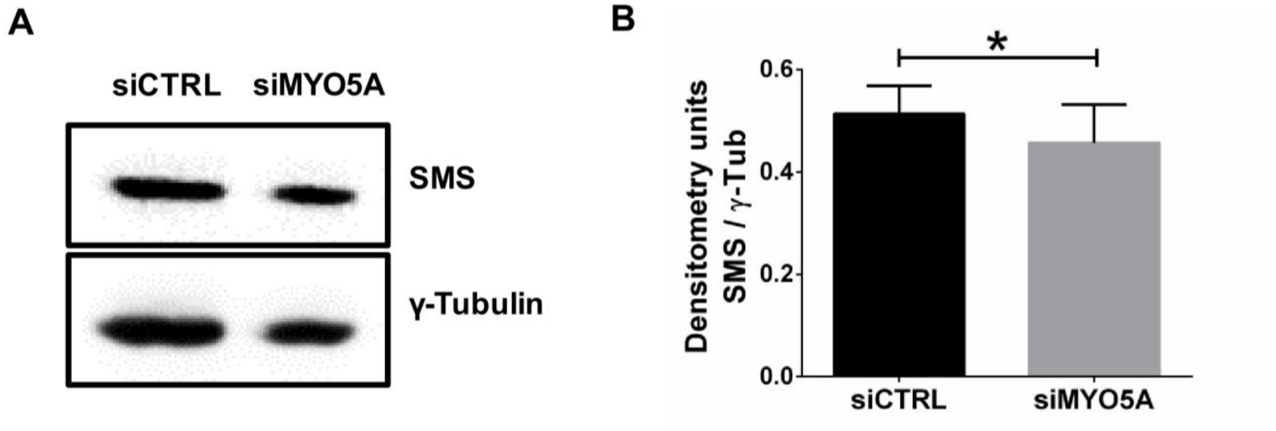


Figure S4. Quantification of SMS protein by western blot (A) in cells treated with siControl or siMYO5A. The graph (B) represents the quantification of the signal intensity of the bands by densitometry, using γ-tubulin as endogenous control. * $p \leq 0.05$, Paired t-test, two-tailed.

Supplementary method

*Western blotting*

MCF-7 cells were plated and transfected in 6-well plates using DharmaFECT 1 Transfection Reagent (GE Healthcare) according to the manufacturer's instructions, with the Stealth RNAi™ siRNA targeting *MYO5A* and the scramble sequence Stealth RNAi™ siRNA Negative Control High GC Duplex (Invitrogen), as previously described. Protein lysates were extracted using RIPA buffer (Tris-HCl 50mM pH 8.0, NaCl 150mM, 1% *(v/v)* Triton X-100, 0,5% *(m/v)* Sodium deoxycholate, 0,1% *(m/v)* sodium dodecyl sulfate), containing Protease Inhibitor Cocktail (Sigma-Aldrich). 20 µg of protein from whole-cell lysates were fractioned by 10% SDS-PAGE and transferred to a nitrocellulose membrane (Bio-Rad). The membranes were incubated with blocking solution [3% *(m/v)* BSA, 0.05% *(v/v)* Tween in TBS (50 mM Tris-HCl pH 8, 150 mM NaCl)] for 1 h at room temperature, and then blotted with the following antibodies: rabbit anti-SMS (Sigma-Aldrich: HPA029852) and mouse anti-ɣ-tubulin (Sigma-Aldrich: T6557) diluted according to manufacturer's instructions in TBS plus 0.05% *(v/v)* Tween and incubated for 2 hours at room temperature. HRP-conjugated secondary antibodies, anti-mouse IgG (Promega: W4021) and anti-rabbit IgG (Promega: W4011), were diluted according to manufacturer's instructions in TBS plus 0.05% *(v/v)* Tween and incubated for 1 hour at room temperature. Detection was made by chemiluminescence on the ChemiDoc XRS+ (Bio-Rad).
